# Association of *PD-1* gene with outcome of hepatitis C virus infection

**DOI:** 10.17179/excli2018-1394

**Published:** 2018-09-24

**Authors:** Jamal Sarvari, Razieh Dowran, Seyed Younes Hosseini, Mohammad Reza Fattahi, Nasrollah Erfani

**Affiliations:** 1 Gastroenterohepatology Research Center, Shiraz University of Medical Sciences, Shiraz, Iran; 2 Department of Bacteriology and Virology, School of Medicine, Shiraz University of Medical Sciences, Shiraz, Iran; 3 Department of Immunology and Institute for Cancer Research, School of Medicine, Shiraz University of Medical Sciences, Shiraz, Iran

**Keywords:** PD-1 gene, polymorphisms, HCV infection, chronic infection

## Abstract

Primary hepatitis C virus infection might be spontaneously cleared or become chronic. Polymorphisms in immune regulatory genes might influence the outcome. The aim of this study was to determine the frequency of genotypes and alleles of *PD-1.3* and *PD-1.5* gene loci in HCV infected patients and their association with the disease outcome. In this study 167 patients with chronic hepatic C and 42 individuals whose infection was spontaneously cleared, and a healthy control group comprising of 300 participants were included. The presence of chronic or spontaneously cleared infection amongst the participants was determined in advance by serologic and molecular methods. Genomic DNA was extracted using salting out method. *PD-1* gene polymorphisms assay was performed using PCR-RFLP method. The frequency of alleles of *PD-1.3* gene locus was significantly higher in the spontaneously cleared HCV infected group (P = 0.03) as well as the healthy control group (P = 0.04) in comparison to the chronic infected participants. In the case of *PD-1.5* locus, there was no association between the frequency of inherited genotype or alleles and HCV infection outcome amongst the three groups. Haplotype analysis showed no statistically significant differences in the frequencies of different haplotypes between the three studied groups. Our finding collectively inferred that individuals with A allele at *PD-1.3* locus might clear HCV infection more frequently than those with T allele. Instead, polymorphisms at *PD-1.5* locus as well as haplotypes emerged from *PD-1.3* G/A and *PD-1.5* C/T might not be significant in the HCV infection outcome.

## Introduction

Since the discovery of Hepatitis C virus (HCV) three decades ago, it still remains as a challenging health issue worldwide ((Alavian et al., 2016[1]). More than 130 million people are infected with this virus and 3-4 million newly infected cases are reported annually throughout the world (Gower et al., 2014[10]). The rate of chronicity vs. spontaneously clearance after HCV exposure ranges 55-85 % and 45-15 %, respectively. Chronic HCV infection might lead to end stage liver diseases, such as liver cirrhosis and hepatocellular carcinoma in 20-50 % of infected cases. The prevalence of HCV infection amongst the Iranian population is estimated to be less than 0.5 % (Hajarizadeh et al., 2016[12]). 

Several factors, including genetic background of the host, age, gender, immunity disorder, viral genotype, transmission route and co-infections with human immunodeficiency virus or hepatitis B virus might influence the HCV infection outcome (Yahoo et al., 2011[30]; Fedorchenko et al., 2010[6]). Amongst the host genetic factors, immune system, including cytokine as well as co-stimulatory molecules might be associated with HCV infection outcome (Sarvari et al., 2017[21]). 

While the adaptive immunity plays a critical role in the pathogenesis of hepatitis C infection, T cell response is vital in eliminating virus infected hepatocyte (Urbani et al., 2006[24]). Accordingly, it was reported that positive and negative regulatory signals that modulate the antigen-specific T cell function are highly associated with the outcome of HCV infection (Wherry et al., 2007[27]). As part of regulatory pathways, PD-1/PD-L1 molecules primarily inhibit the virus-specific CD8 cell function in viral infections (Barber et al., 2006[2]). Studies have shown that the PD-1 over-expression and resultant specific T cells dysfunctions in acute hepatitis C virus infection can cause significant impairment in cell mediated immunity, followed by HCV persistence. Anti-PDL-1 antibody retrieves HCV-specific CD8+ cells function by blocking the inhibitory PD-1/PD-L1 pathway. This can be employed as an immunotherapeutic approach for chronic HCV infection (Fuller et al., 2013[8]; Urbani et al., 2008[25]).

Moreover, scientists have shown that exhaustion and anergy of CD8+ T cells following over-expression of the inhibitory receptors like PD-1, is significantly amplified in individuals with persistent HCV infection (Larrubia et al., 2011[15]). Also, Xiao et al. and others have shown that HCV infection might be involved in impairment of CD8+ and CD4+ cell responses and HCV core and F proteins induced T cell dysfunction by interacting with PD-1/PD-L1 pathway (Xiao et al., 2016[28]; Yao et al., 2007[31]).

Hence, it has been proposed that over-expression of inhibitory receptor PD-1 on lymphocyte lineages is associated with poor HCV-specific T cell responses and chronic infection (Bowen and Walker, 2005[4]; Fuller et al., 2010[9]). On the other hand, reduced expression of PD-1 is correlated with appropriate HCV-specific CD4+ and CD8+ T cells response, and spontaneous clearance of HCV infection (McMahan et al., 2010[16]).

The PD-1, also known as CD279 gene is located on chromosome 2q37.3 (Riella et al., 2012[20]). It is an immunoglobulin super family member, which acts as a CD28 regulatory molecule (Haghshenas et al., 2011[11]; Mojtahedi et al., 2012[17]). PD-1 was originally identified in a T cell line undergoing activation induced cell death (AICD), and is expressed on the surface of activated T and B cells as well as myeloid cells (Vibhakar et al., 1997[26]). After T cells activation, PD-1 over-expression performs inhibitory effects in order to regulate the duration and extent of their actions (Freeman et al., 2000[7]). After PD-1 interaction with its cognate ligands PD-L1 (B7-H1) and PD-L2 (B7-DC), proliferation and cytokine production by CD4 and CD8 T lymphocytes is inhibited, and tissue inflammation/damage reduced (Freeman et al., 2000[7]).

It has been reported that several* PD-1 *single nucleotide polymorphisms (SNPs) attribute to disease progression (Prokunina et al., 2002[19]; Kroner et al., 2005[14]; Bertsias et al., 2009[3]), of which, *PD-1.3* G/A (+7146, dbSNPrs # cluster id: rs11568821, in intron 4) and *PD-1.5* C/T (+7785 or +872, dbSNPrs # cluster id: rs2227981, in exon 5) has special importance (Prokunina et al., 2002[19]; Kroner et al., 2005[14]; Bertsias et al., 2009[3]). The role of *PD-1* polymorphisms on viral diseases is of an importance (Zheng et al., 2010[36]; Xiao et al., 2015[29]; Zhang et al., 2012[33]), but only few publications have investigated the role of *PD-1* polymorphisms, especially in HCV infection outcomes (Xiao et al., 2015[29]). 

Based on the inhibitory role of PD-1 in immune system, and the association of its expression with SNPs, we took the liberty to investigate *PD-1* gene in genetic susceptibility of individuals to Hepatitis C outcome. To the best of our knowledge, data regarding the correlation between polymorphisms and the outcome of HCV infection are limited. Moreover, controversial results suggest the role of PD-1 expression in predicting the disease outcome (Kasprowicz et al., 2008[13]). Accordingly, the aim of this study was to determine the frequency of *PD-1* gene polymorphisms at *PD-1.3* G/A and *PD-1.5 *C/T loci in Iranian patients and its association with HCV infection outcome (chronicity versus clearance).

## Subjects and Methods

A total of 209 patients were recruited consecutively from the Gastroenterohepatology research center at Nemazee Hospital in Shiraz, Iran from September 2012 to March 2015. Out of which, 42 patients was resolved spontaneously from HCV infection, and 167 patients had chronic HCV. A control group consisting of 300 healthy individuals was included in the study. The study was approved by the local Ethics committee of Shiraz University of Medical Sciences, and written informed consent was obtained from each participant before sampling.

### Viral infection state

The status of HCV infection (Chronic or spontaneously clearance) was determined through patients' medical records, but further confirmed by serological and molecular methods as described previously (Sarvari et al., 2016[22]). Briefly, all sera samples were screened by ELISA assay to confirm serological state. Two separate qualitative PCR methods were used to detect viral genome. An in-house Nested-PCR method was used primarily as a screening method. To make sure, a commercial qualitative Real-Time PCR (Amplisens HCV-FRT, Russia) was also employed to detect the viruses. 

### Genomic DNA extraction and analysis of cytokine polymorphisms 

A blood sample of 5 ml was drawn from each participant and mixed in EDTA anticoagulant. DNA was extracted from peripheral blood leukocytes using the salting out procedure. The quality and quantity of the extracted DNA was examined by a Nanodrop^TM ^densitometry. Polymorphisms at *PD-1.3* G/A (+7146) and *PD-1.5* C/T (+7785) positions were identified using polymerase chain reaction-restriction fragment length (PCR-RFLP) (Haghshenas et al., 2011[11]). The PCR reaction was performed in total volume of 15 μl, containing 1X reaction buffer, 200 μm of each dNTPs (Cinnagene Inc. Iran), 1 U Taq DNA polymerase (Cinnagene Inc. Iran), 0.5 μM of each specific primers 200-300 ng of DNA template and 0.4 and 0.3 mM MgCl_2_for *PD-1.3* G/A and *PD-1.5* C/T loci, respectively. Afterward, all positive samples were introduced into *PstI *and *PvuII* enzymes digestion to find polymorphisms at *PD-1.3* G/A(+7146) and *PD-1.5* (+7785 C/T). The digestion pattern of each reaction was developed after the products ran on 2 % agarose gel.

### Statistical methods 

The results were analyzed by χ2 test using Epi info 2000 (CDC, USA) and SPSS software package version 11.5 (SPSS Inc, Chicago, IL, USA). Haplotype estimation was evaluated by Arliquin 3.1 software package (Excoffier et al., 2005[5]). P-values less than 0.05 were considered to be statistically significant.

## Results

A total of 209 subjects infected with HCV were enrolled in this study. The outcome of HCV infection in 167 individuals was determined as chronic status, while the reset had spontaneously cleared infection. Male to female ratios in chronic and spontaneous cleared groups were 143/24 and 38/4, which were not significantly different (P = 0.56). 

The polymorphisms of *PD-1* at +7146 G/A (*PD-1.3*) and +7785 C/T (*PD-1.5*) loci were determined in 203 and 193 individuals in HCV groups. The distribution of PD.1 genotype and allele at *PD-1.3* G/A and *PD-1.5* C/T loci in cleared, chronic and control groups is shown in Tables 1(Tab. 1), 2(Tab. 2) and 3(Tab. 3). Statistically significant difference was only seen in allele distribution of PD1.3 between spontaneously cleared and the control group in comparison with chronic infection group.

The frequency of A allele of PD1.3 locus was higher in those whose infection was cleared (13.9 % vs. 6.3 %) compared to subjects with chronic HCV infection. Allele A frequency of PD1.3 locus was higher in the control group (11 % vs. 6.9 %) in comparison to chronic HCV infection. PD1.3 genotypes were not significantly different among the three studied groups. In the case of PD1.5 locus, no association was observed between frequency of genotype and allele amongst the three studied groups.

The study analysis showed that these polymorphisms were under linkage disequilibrium. Distribution of different haplotype frequencies in cleared, chronic and control groups is shown in Table 4(Tab. 4), 5(Tab. 5) and 6(Tab. 6). The frequency of four different haplotype including GT, GC, AC and AT was not statistically significant amongst the three study groups.

## Discussion

A number of factors including HCV genotype, age, gender, degree of liver fibrosis, alcohol consumption, co-infections with HIV/HBV and genetic background presumed to be associated with HCV outcome (Yee, 2004[32]). Amongst them, genetic background exhibits a special role in determining the fate of HCV infection. In this regards, certain immune regulatory elements, such as PD-1 is associated with chronic viral hepatitis (Urbani et al., 2006[24]; Xiao et al., 2015[29]; Zhang et al., 2014[34]). 

We previously showed that polymorphisms in IFN-γ and IL-28B gene are associated with response to therapy, as well as spontaneously clearance state in HCV infection (Sarvari et al., 2014[23], 2016[22], 2017[21]). In the current study we investigated the impact of *PD-1* gene polymorphisms on HCV infection outcome.

The results revealed that the frequency of A allele at *PD-1.3* G/A(+7146) locus in individuals whom HCV infection spontaneously cleared and healthy control group was higher than those with chronic infection. In this regards, Zhang et al. (2014[34]) reported that patients with HBV chronic infection including those with chronic active hepatitis and hepatocellular carcinoma had significantly elevated PD-1 mRNA levels in comparison with the healthy controls.

Also, Urbani et al. (2008[25]) and Fuller et al. (2013[8]) reported that anti-PDL-1 antibody can improve HCV-specific CD8 cells function by blocking PD-1/PD-L1 pathway. In addition, Larrubia et al. (2011[15]) showed that inhibitory receptor like PD-1 expression increase in HCV persistence infection, which might be related to the exhaustion and anergy of CD8+ T cells. A Low/controlled expression of PD-1 in those with spontaneously cleared HCV infection might be associated with the presence of effective HCV-specific CD4+ and CD8+ T cells, which consequently provides protective immunity to combat virus replication in the acute phase (McMahan et al., 2010[16]). On the contrary, high level expression of this molecule might be associated with reduced cytokine production, proliferation and cytotoxicity impairments of virus-specific CD8+ T cell and viral persistent status (Bowen and Walker, 2005[4]; Fuller et al., 2010[9]). This is in accordance with the result of a study in Italy suggesting that increased amount of virus in patients with chronic HCV infection are associated with enhanced expression of PD-1 on virus-specific CD8+ cells (Urbani et al., 2008[25]). These findings also suggested that the level of PD-1 expression is partially related to PD1.3 polymorphism.

A possible hypothesis for the role of *PD-1.3* G/A polymorphism in diseases such as HCV infection is the result of substituting A for G in the enhancer region (Prokunina et al., 2002[19]). This type of substitution, disrupts the binding site of Runt-related transcription factor-1 (RUNX-1, also called AML-1), which might down-regulate the relevant gene expression. Low expression of PD-1 molecule in individuals who carry A allele leads to the impairment of its inhibitory effect and retention of lymphocyte activity (Haghshenas et al., 2011[11]; Kroner et al., 2005[14]).

Regarding PD-1 polymorphisms effect on the other viral diseases progression, Niknam et al. (2016[18]) reported a significantly higher frequency of GG genotype and G allele of PD-1.3A/G polymorphism in kidney transplant patients infected with reactivated cytomegalovirus. Moreover, it was reported that there was no significant difference in the frequencies of genotypes and alleles at PD-1.3 between breast cancer patients and healthy individuals (Haghshenas et al., 2011[11]).

When the frequency of different genotype of PD-1.3 loci was analyzed, there was no statistical significant association between the frequency of genotype in individuals whose HCV infection was cleared with those who had chronic HCV infection as well as healthy control groups.

Similarly, by looking at the frequency result of *PD-1.5* (+7785 C/T), it revealed no significant differences in genotypes and alleles amongst the three investigated groups. In line with our study, Haghshenas et al. (2011[11]), reported no significant association in the frequencies of genotypes and allele of *PD-1.5* with breast cancer. However, Mojtahedi et al. (2012[17]) reported that *PD-1.5* C/T (+7785) polymorphism was associated with colon cancer progression in Iranian population.

Other studies investigated the role of other PD.1 polymorphisms in viral hepatitis outcomes. In this regard, Xiao et al. (2015[29]) showed that PD1.6 TT genotype was associated with an increased risk of HCV infection chronicity. They reported that the C allele of PD1.6 might be involved in protecting females from persistent infection whereas a higher proportion of PD-1 expression on T cells was observed in TT genotype in comparison to CC genotype (Xiao et al., 2015[29]). In addition, Zhang et al. (2010[35]) reported *PD-1.6* GG genotype and G allele might be involved in the down-regulation of PD-1 expression were less frequent in HBV patients compared to the healthy control group. But in the case of *PD-1.1*, they showed no difference in the genotype and allele frequencies amongst those with chronic HBV infection and the healthy control group (Zhang et al., 2010[35]). Also, it was reported that none of P7209C/T and the P8737A/G polymorphism sites were associated with the establishment of chronic HBV infection (Zheng et al., 2010[35]). 

Haplotype analysis is a useful method for discovering predictive genes, which might be involved in diseases progression (Sarvari et al., 2016[22]; Haghshenas et al., 2011[11]; Zhang et al., 2010[35]). In the mentioned study, haplotypes were determined in order to find out the significant effect of different haplotypes on HCV infection outcome. 

Investigating the frequency of four different haplotype emerged from *PD-1.3* G/A and *PD-1.5* polymorphisms (GT, GC, AC and AT) revealed that there was not statistically significant association amongst the three studied groups. In the case of other diseases, no difference in the frequency of four different haplotypes were observed between breast cancer patients and healthy controls (Haghshenas et al., 2011[11]). Regarding *PD-1.1* and *PD-1.6* polymorphism, Zhang et al. (2014[34]) reported that *PD-1.1*G/*PD-1.6*G haplotypes were less frequent in patients with chronic HBV than the controls. 

## Conclusion

In conclusion, according to the higher frequency of A allele of *PD-1.3* G/A (+7146) loci in those with spontaneously cleared HCV infection and the healthy control group, we concluded that individuals with this genetic background might be more resistant to HCV infection than those with T allele at this locus. Also, our results showed that other genotype/allele and haplotypes of *PD-1.3* G/A and *PD-1.5* C/T were not associated in determining the hepatitis C infection outcome.

## Acknowledgement

We appreciate the collaboration of our colleagues in the Department of Bacteriology and Virology as well as the Institute for Cancer Research (ICR) at Shiraz University of Medical Sciences. We also would like to thank Mrs. Maryam Mansurabadi for sampling the patients. This work was supported by grants from Shiraz University of Medical Sciences, Shiraz, Iran (Grant No.93**-**7259). The authors wish to thank Mr. H. Argasi at the Research Consultation Center (RCC) of Shiraz University of Medical Sciences for his invaluable assistance in editing this manuscript.

## Conflict of interest

The authors declare no conflict of interest.

## Figures and Tables

**Table 1:**
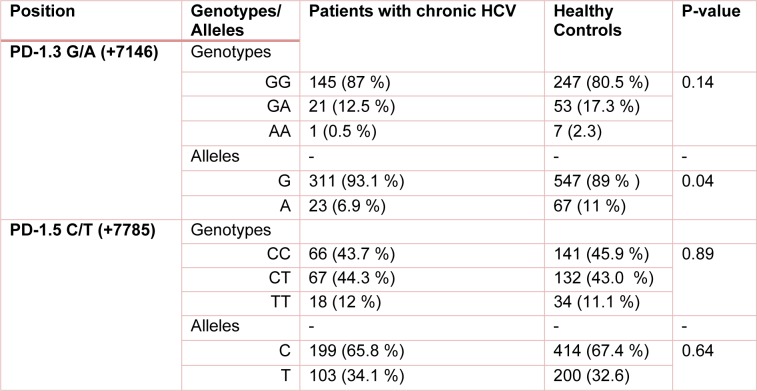
The frequency of *PD-1* genotypes/alleles in patients with chronic HCV infection and healthy control group. P-value less than 0.05 was considered to be statistically significant.

**Table 2:**
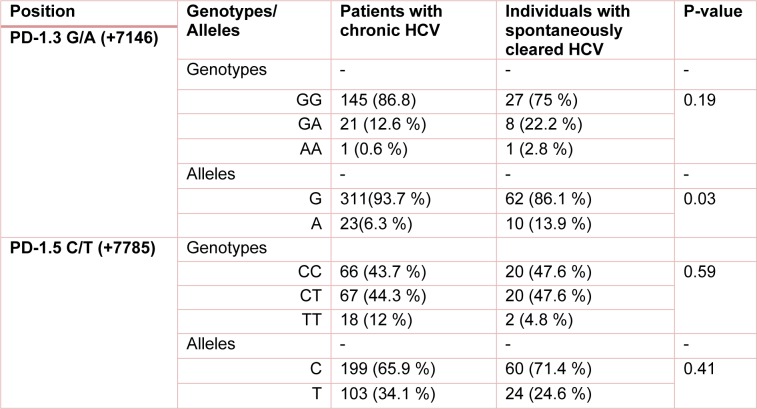
The frequency of PD1 genotypes/alleles in patients with chronic HCV and individuals with spontaneously cleared HCV. P-value less than 0.05 was considered to be statistically significant.

**Table 3:**
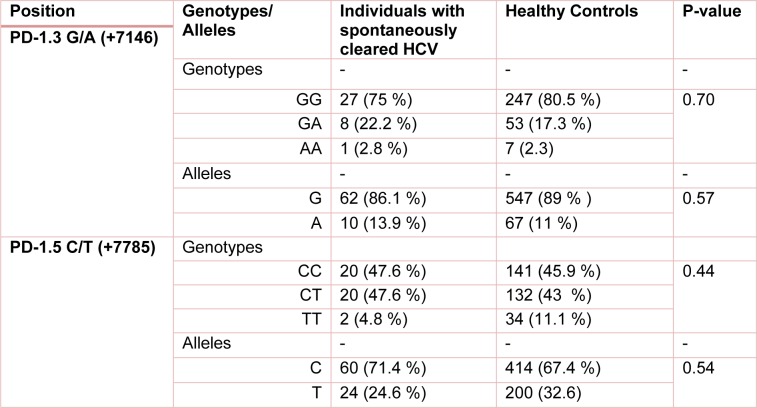
The frequency of PD1 genotypes/alleles in individuals with spontaneously cleared HCV and healthy controls. P-value less than 0.05 was considered to be statistically significant.

**Table 4:**
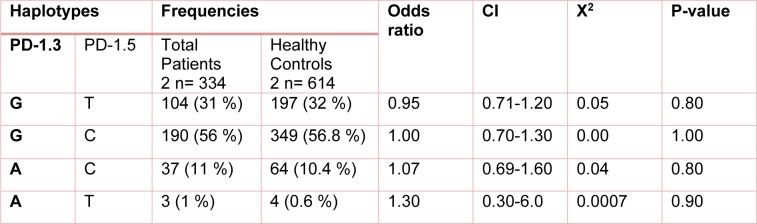
Distribution of the estimated haplotype frequencies emerged from PD-1.3 G/A (+7146) and PD-1.5 C/T (+7785) in total patients (those with chronic HCV or spontaneously cleared HCV) and healthy control group. CI; 95 % confidence Interval

**Table 5:**
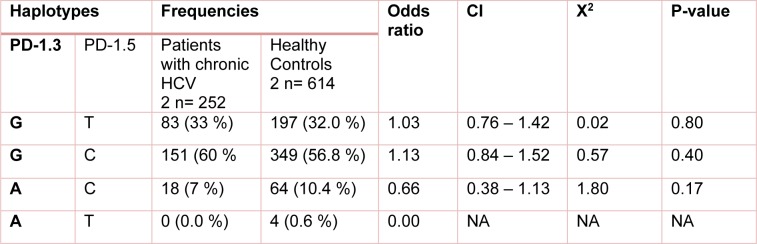
Distribution of the estimated haplotype frequencies emerged from PD-1.3 G/A (+7146) and PD-1.5 C/T (+7785) in patients with chronic HCV and healthy control group. CI; 95 % confidence Interval, NA; Not applicable

**Table 6:**
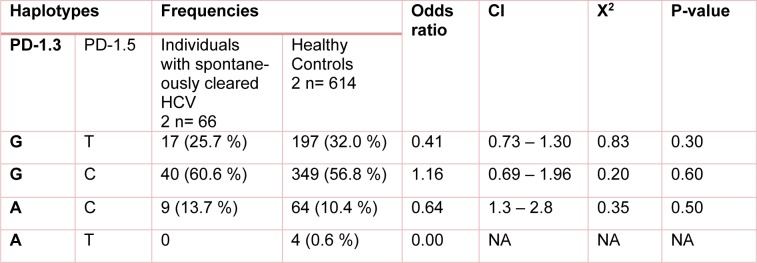
Distribution of the estimated haplotype frequencies emerged from PD-1.3 G/A (+7146) and PD-1.5 C/T (+7785) in individuals with spontaneously cleared HCV and healthy control group. CI; 95 % confidence Interval, NA; Not applicable
